# Digital multi-modal approaches to subtyping insomnia disorder (DIMOSI): study design, rationale, digital platform, and preliminary baseline characteristics of a national prospective cohort study

**DOI:** 10.1186/s12888-025-07648-9

**Published:** 2025-12-10

**Authors:** Yujing Zhou, Jiyang Pan, Hanrong Cheng, Li Xiao, Wenjing Zhang, Hui Huang, Kezhi Liu, Leqin Fang, Wenbin Ma, Yan Xia, Jinghui Li, Dongsheng Lv, Yanyu Hu, Yi Chang, Zan Wang, Haojuan Tao, Chunrong Zhang, Chenyu Li, Yanhui Peng, Qiying Zhao, Yunshu Zhang, Junhua Mei, Xuehang Wang, Ting Wei, Mingqing Zhou, Yi Zhang, Qiuqiang Chen, Ngan Yin Chan, Bin Zhang, Yun Kwok Wing, Binbin Lei, Jihui Zhang

**Affiliations:** 1 Longgang Maternity and Child Institute of Shantou University Medical College (Longgang District Maternity & Child Healthcare Hospital of Shenzhen City), Shenzhen, China; 2 Center for Sleep and Circadian Medicine, The Affiliated Brain Hospital, Guangzhou Medical University, Guangzhou, Guangdong China; 3 Guangdong Engineering Technology Research Center for Translational Medicine of Mental Disorders, Guangzhou, China; 4 The First Affiliated Hospital, Jinan University 613 West Huangpu Avenue, Guangzhou, 510630 China; 5 Department of Sleep Medicine, Institute of Respiratory Diseases, Shenzhen People’s Hospital, The Second Clinical Medical College of Jinan University, The First Affiliated Hospital of Southern University of Science and Technology, Shenzhen, Guangdong China; 6 Sleep Medicine Center, Shengjing Hospital of China Medical University, Shenyang, Liaoning 110004 China; 7 Shanghai Municipal Hospital of Traditional Chinese Medicine, Shanghai University of Traditional Chinese Medicine, Shanghai, 200071 China; 8 Department of Sleep Disorders, Wuhan Mental Health Center, Wuhan, Hubei 430012 China; 9 Department of Psychiatry, Fundamental and Clinical Research on Mental Disorders Key Laboratory of Luzhou, Medical Laboratory Center, Laboratory of Neurological Diseases & Brain Function, Affiliated Hospital of Southwest Medical University, Luzhou, Sichuan China; 10 Department of Psychiatry, Sleep Medicine Center, Nanfang Hospital, Southern Medical University, Guangzhou, China; 11 Department of Neurology, Binzhou Medical University Hospital, No. 661 Huanghe 2nd Road, Binzhou, Shandong 256600 China; 12 1st Affiliated Hospital of Harbin Medical University, 23 Youzheng Street, Harbin, Heilongjiang China; 13 Shiyan Renmin Hospital, Shiyan, China; 14 Inner Mongolia Autonomous Mental Health Center, Hohhot, China; 15 Xiamen Xianyue Hospital, Xianyue Hospital Affiliated with Xiamen Medical College, Fujian Psychiatric Center, Fujian Clinical Research Center for Mental Disorders, Xiamen, China; 16 The First Affiliated Hospital of Dalian Medical University, 222 Zhongshan Road, Xigang District, Dalian, Liaoning China; 17 The First Bethune Hospital of Jilin University, Xinmin Street 1, Chang Chun, 130021 China; 18 Department of Psychiatry, National Clinical Research Center for Mental Disorders, and National Center for Mental Disorders, The Second Xiangya Hospital of Central South University, Changsha, 410011 China; 19 Qinhuangdao Haigang Hospital, Heibei, China; 20 Sleep Center, Department of Neurology, Chongqing Hospital of Traditional Chinese Medicine, Chongqing, 400021 China; 21 Sixth Affiliated Hospital of Xinjiang Medical University, No.39, Wuxing South Road, Tianshan District, Urumqi, Xinjiang China; 22 Xinjiang Changji Hui Autonomous Prefecture People’s Hospital, No. 303, Yan’an North Road, Changji City, China; 23 The Sixth People’s Hospital of Hebei Province, Baoding, Hebei China; 24 Wuhan No.1 Hospital, Wuhan, Hubei China; 25 International Education School of China Medical University, No.77 Puhe Road, Shenyang, Liaoning 110122 China; 26 Li Chiu Kong Family Sleep Assessment Unit, Department of Psychiatry, Faculty of Medicine, The Chinese University of Hong Kong, Hong Kong, China; 27 Department of Psychiatry, Faculty of Medicine, The Chinese University of Hong Kong, New Territories, Hong Kong, China; 28 School of Public Health and Management, Guangzhou University of Chinese Medicine, Guangzhou, 510006 China; 29 Guangdong Mental Health Center, Guangdong Provincial People’s Hospital (Guangdong Academy of Medical Sciences), Southern Medical University, Guangzhou, China

**Keywords:** Insomnia disorder, Multi-modal approaches, Subtyping, Prospective study, Digital platform

## Abstract

**Background:**

Insomnia disorder exhibits complex manifestations and heterogeneous clinical trajectories. Accurate subtyping of insomnia might enhance understanding of its clinical presentations and facilitate precision management. The Digital Multi-modal approaches to Subtyping Insomnia disorder (DIMOSI) study is a national prospective cohort study utilizing multi-modal assessments to explore the subtypes of insomnia disorder, their natural trajectories, and related mental health outcomes.

**Methods:**

A total of 4,000 adult participants meeting International Classification of Sleep Disorders, 3rd Edition (ICSD-3) criteria for insomnia disorder will be recruited from community or clinical settings. Eligible participants will be invited to complete the multi-dimensional assessments via a digital platform, including a structured interview, questionnaires, cognitive tasks, sleep-activity diary, physiological characteristics, and ecology momentary assessments, as well as 7-day physical activity and sleep tracking using wearable devices. All participants will be followed up at 6 and 12 months. The primary outcome is the identification of multi-modal subtypes of insomnia disorder and their correlates. Secondary outcomes include the longitudinal trajectories of these subtypes, associated risk factors, and mental health outcomes.

**Results:**

As of June 30, 2025, a total of 2937 patients with insomnia disorder have been recruited, with a mean age of 37.3 years (SD = 12.6), 59.3% from outpatient clinics, and 66.5% female. Among the participants, 2850(97.1%) were suffering from current insomnia disorder, while the mean score of the ISI was 15.5 ± 5.8. A total of 2134 participants (72.7%) wore accelerometers, while 2429 (82.7%) wore wearable EEG monitors for continuous assessments.

**Discussion:**

The DIMOSI study is a large-scale national prospective cohort investigating insomnia disorder utilizing a self-developed digital multi-modal platform. It integrates comprehensive subjective and objective assessments from 33 centers in China. The current study offers a unique opportunity to explore subtypes of insomnia disorder and their natural course and their correlates through the digital multi-modal platform that provides enriched and comprehensive assessments. It may provide the potential to inform the development of personalized prevention and intervention strategies, ultimately improving patient outcomes.

**Trial registration:**

Clinical Trial Registry Name: Digital Multi-modal approaches to deep phenotyping insomnia disorder. Registration Number: ChiCTR2200056425. Date of Registration: 2022-02-05.

**Supplementary information:**

The online version contains supplementary material available at 10.1186/s12888-025-07648-9.

## Background

Insomnia symptoms affect 19%-50% of adults globally [1], with approximately 10% meeting the criteria for insomnia disorder [2]. Patients with insomnia disorder often experience impaired quality of life and general health [2], along with various adverse outcomes, including coronary heart disease, depression, and increased risk of self-harm and suicidality [3, 4]. Insomnia disorder imposes a significant burden not only on patients but also on their families and society [5, 6]. Community-based studies have shown that the 5-year persistence rate for insomnia symptoms ranges from 32.6% to 42.5% [7]. Even with adequate treatment, such as pharmacotherapy or cognitive behavioral therapy for insomnia (CBTI), approximately 20%-40% of patients respond poorly, likely due to heterogeneity in pathogenesis and clinical presentations [8–10]. Therefore, to overcome the limitations in the assessment and treatment of insomnia disorder, it is crucial to establish accurate subtypes based on comprehensive assessments for its core features and related characteristics [11, 12]. Multi-modal subtyping insomnia disorder could empower the development of sleep medicine by informing not only insomnia diagnosis and symptomatic management but also prognostic prediction and personalized intervention selection.

Significant efforts have been made in recent decades to clarify the diverse manifestations of insomnia disorder. First, the most commonly used classification in general clinical practice is based on nocturnal complaints [2] according to the International Classification of Sleep Disorders - Third Edition (ICSD-3) [7, 13]. Clinicians typically prescribe hypnotics with different half-lives based on difficulty initiating sleep (DIS), difficulty maintaining sleep (DMS), or early morning awakenings. However, over half of patients with insomnia disorder report at least two types of nocturnal symptoms, and the stability of this classification system is often unsatisfactory [8, 14]. Second, Vgontzas and colleagues have found that insomnia with objectively short sleep duration as measured by a single-night nocturnal polysomnography (PSG), may represent a more biologically severe subtype with a diminished response to CBTI compared to insomnia without short sleep duration [15]. However, PSG requires overnight hospitalization, which is costly, time-consuming, and limits scalability [16]. Third, recent studies have attempted to use emotional processing and cognitive functions to subtype insomnia disorder [12], but their application in clinical practice remains underexplored. Finally, a recent effort using portable sleep assessments extracted insomnia-related subtypes from a large-scale cohort, but clinical interpretations of these subtypes were not provided [17].

Taken together, previous studies have proposed several subtypes of insomnia disorder based on subjective and objective measures [18–20]. However, subtyping methods that rely solely on subjective reports are easy to implement but will lack objective indicators, resulting in ambiguous classifications. On the other hand, subtyping based on PSG monitoring is limited in capturing the temporal and spatial fluctuations of sleep in insomnia. Moreover, the current classification schemes for insomnia provide limited guidance for clinical treatment [21]. This classification challenge is closely tied to the high heterogeneity of insomnia disorder. In the current study, we plan to integrate the data of sleep and circadian rhythm, mental distress, behavior, and cognitive function, from both subjective reports and long-term ambulatory monitoring.

The rapid advancement of digital technology over the last decade has revolutionized mental health care, providing new opportunities for the assessment and management of mental disorders [22–25]. Wearable devices, including smartphones and accelerometers (Axivity AX3), have emerged as tools to monitor sleep and circadian rhythm, capturing changes in physical activity, environment, and related mental status simultaneously [26, 27]. However, few assessment platforms have integrated subjective and objective measurements, for insomnia disorder. To systematically assess core features of insomnia disorder and its related characteristics, an integrated digital platform, called Zhiyoumian (知优眠 in Chinese, meaning “know and improve your sleep”) was established for this study, consisting of two apps and a website for comprehensive assessments.

**Fig. 1:**
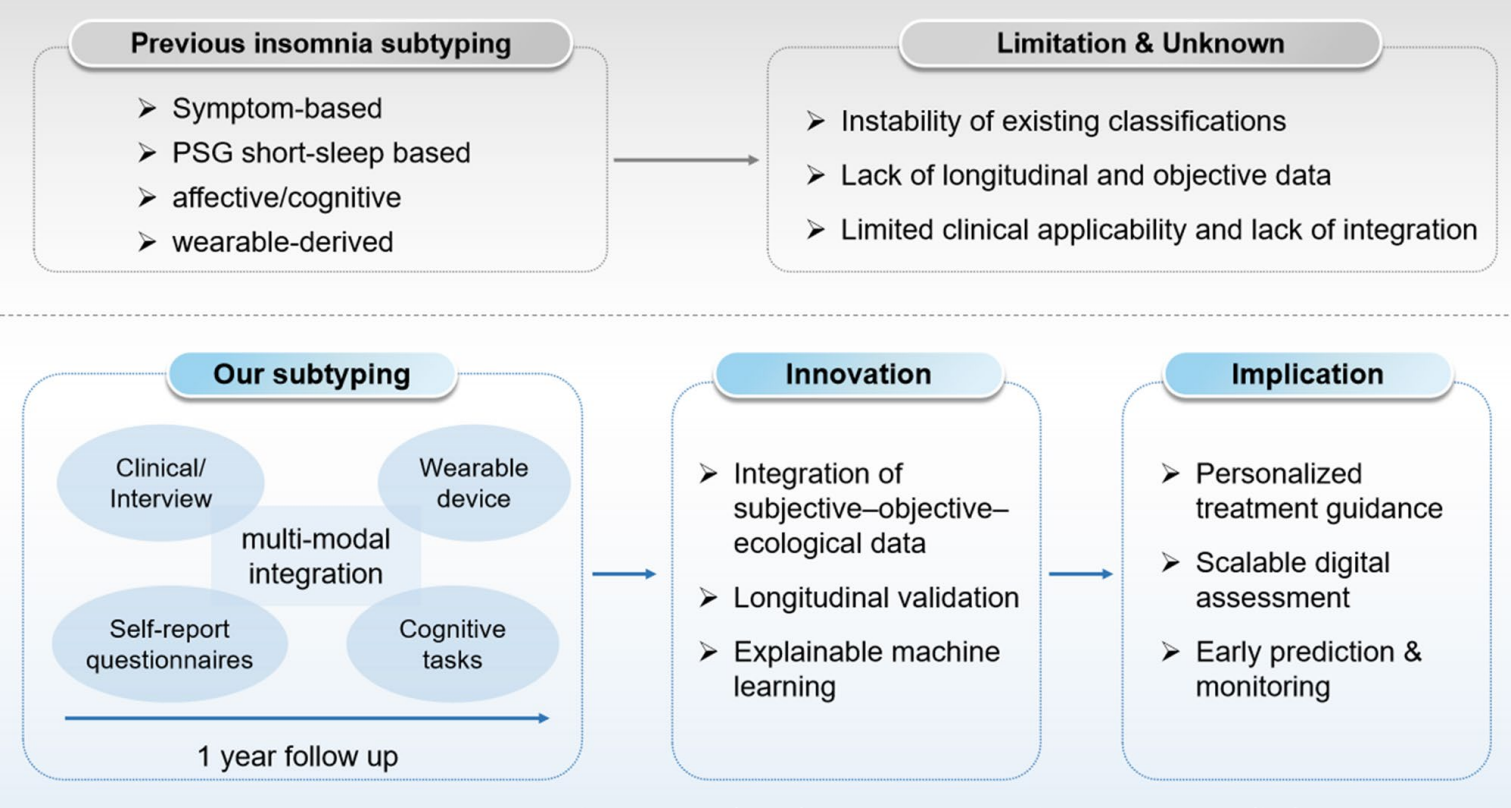
Conceptual model of multimodal digital subtyping for insomnia disorder

The Digital Multi-modal Approaches to Subtyping Insomnia Disorder (DIMOSI) study (ChiCTR2200056425 in Chinese Clinical Trial Registry) is designed to address the critical gaps, including: (a)providing objective and continuous data to complement subjective reports, (b) capturing dynamic, real-world fluctuations rather than the single-night PSG assessment, and (c) simultaneously assessing multiple functional modalities to create a holistic profile. We hypothesize that the integration of multi-modal digital data, including clinical interview, self-report, ecological momentary assessment (EMA), data from wearable devices, and cognitive tasks, may capture the interaction between the behavioral, emotional, circadian, and cognitive systems that underlie insomnia heterogeneity, leading to a precise subtype system of insomnia disorder. The DIMOSI aims to achieve the following: (a)exploring the subtypes of insomnia disorder using comprehensive multi-modal assessment approaches, (b) validating the stability and predictive accuracy of the identified subtypes through a prospective study with follow-up assessments at 6 months and 12 months (c)determining the natural trajectories and related mental health outcomes of different subtypes of insomnia disorder. This fine-grained, data-driven subtyping study for insomnia disorder is anticipated to transcend unstable, symptom-based classifications and accelerate the development of clinical paradigm (Fig. 1).

## Methods

The Digital Multi-modal Approaches to Subtyping Insomnia Disorder (DIMOSI) study is a national prospective cohort study recruiting 4,000 individuals with a lifetime history of insomnia disorder from both clinical and community settings. Clinical setting includes outpatient clinics of psychiatry neurology, and sleep, while the individuals recruited from community health centers, promotional posters, and social media are recognized to be the participants from community setting. Participants will be invited to complete assessments via a digital platform, which includes data collection through smartphones, accelerometers, (Axivity AX3) and the wearable electroencephalogram (EEG) monitors for the comprehensive multi-modal evaluation of insomnia disorder and its related characteristics at baseline and follow-up points.

### Ethic declaration

All procedures in the DIMOSI study will comply with the ethical standards of the relevant national and institutional committees on human experimentation. Ethical approval was obtained from the Ethics Committee of the Guangdong Provincial People’s Hospital (KY-Z-2022–002-01), and each collaborating institution also secured approval from its respective Research Ethics Committee. The current study will be conducted in accordance with the Declaration of Helsinki.

### Consent to participate declaration

Every human participant provides their consent prior to enrollment.

### Study population

To determine the subtypes of insomnia disorder and explore their predictive value for trajectory and intervention outcomes, an estimated 40–80 variables will be included in the statistical model. Based on an estimated events per variable (EPV) of 50 to ensure sufficient statistical power, a total of 4,000 participants are required at the baseline [28].

Participants aged 18–65 years with a lifetime history or current insomnia disorder will be recruited. Subjects will be included in the study if they meet the following inclusion criteria: a. Previously or currently meeting the diagnostic criteria for insomnia disorder in ICSD-3; b. Ability to read and understand Chinese, and use the devices required for the study; c. Ability to provide informed consent. The exclusion criteria are: a. Lifetime history of schizophrenia, intellectual disability, or severe physical illness; b. Pregnancy or lactation; c. current involvement in other clinical research projects.

**Fig. 2:**
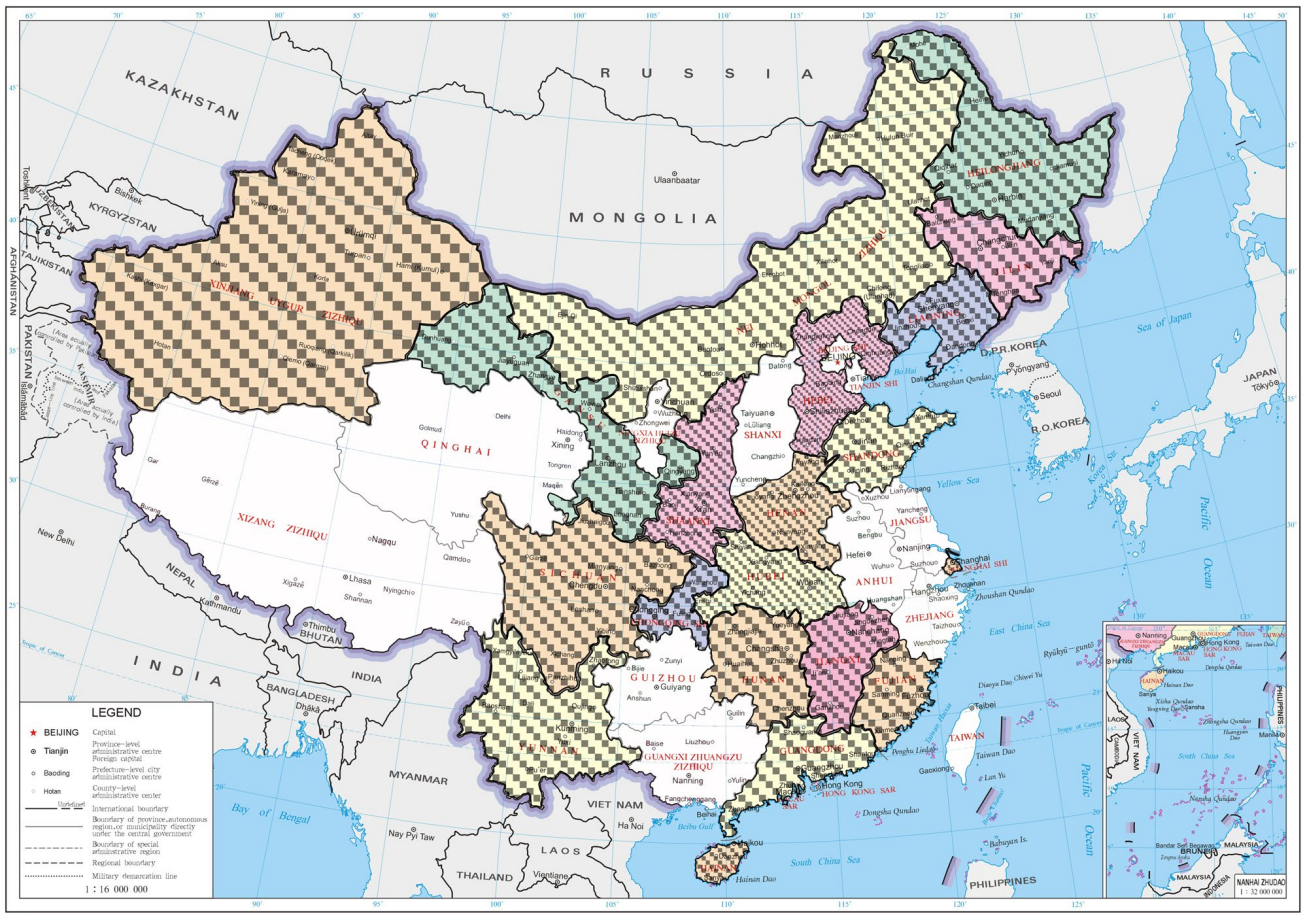
Geographic distribution of the 33 collaborative study sites across China

As of June 2025, 33 study sites specializing in psychiatry, neurology, or sleep medicine have taken part in the current study. The principal research centre of the DIMOSI study is located at the Guangdong Mental Health Centre, Guangdong Provincial People’s Hospital (Guangdong Academy of Medical Sciences), Southern Medical University, Guangzhou, China. Additionally, 33 study sites across various regions of China have joined as collaborative institutes (Fig. 2).

All individuals participating in the study are permitted to continue their ongoing treatments and medications, which will be recorded and documented throughout the research. In addition, electronic Cognitive Behavioral Therapy for Insomnia (e-CBTI), a routine fee-based clinical service delivered via a smartphone application, was optionally accessible to participants; for those who opted in, the study team covered the service fee without modifying the treatment content or delivery.

### Study procedures

**Fig. 3:**
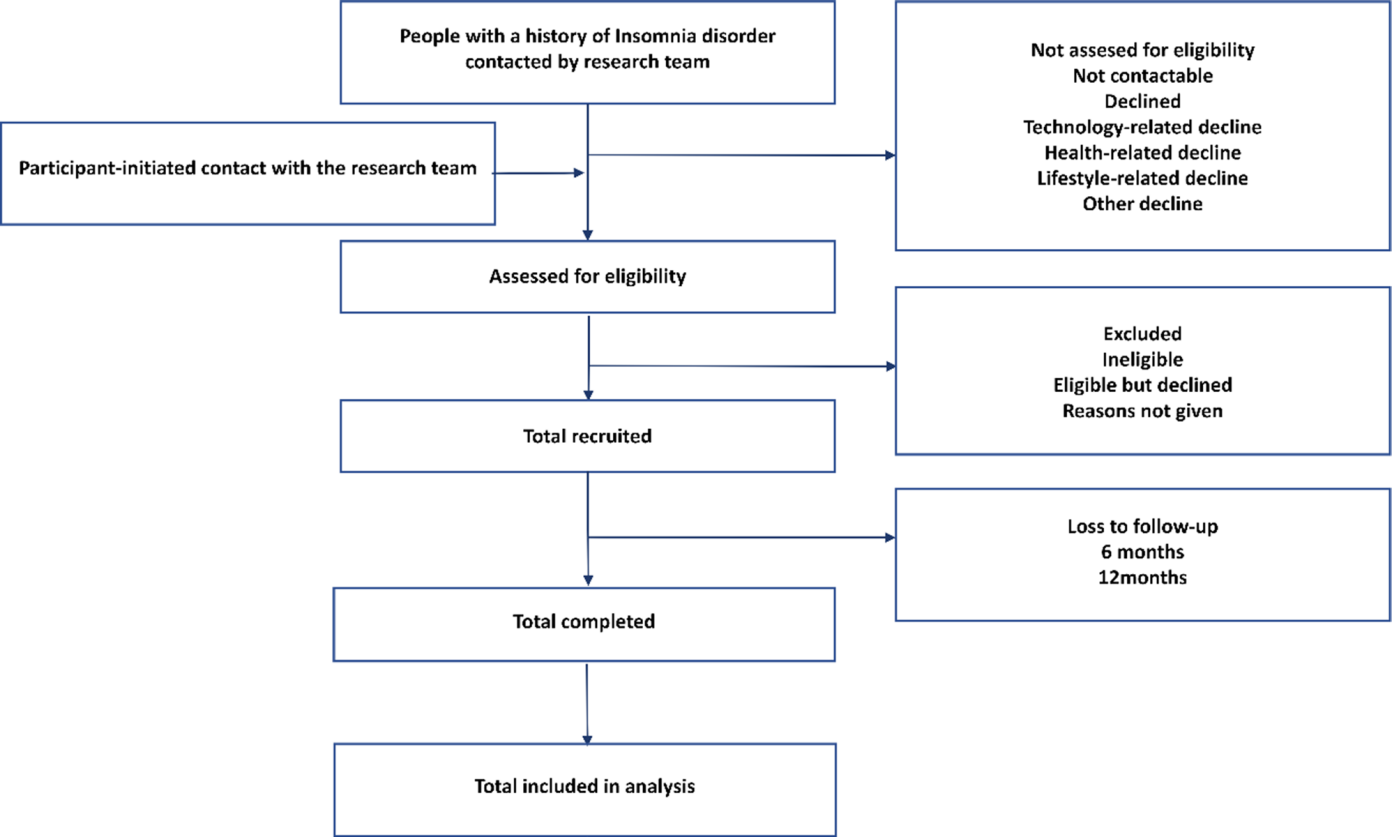
Flowchart of participant recruitment and screening process

Each site adopted systematic convenience sampling, screening for all eligible participants from both community and clinical sources who are willing to join the study. Screening logs and reasons for exclusion are documented to evaluate potential selection bias. Standardized recruitment and assessment procedures are implemented uniformly across all participating sites. Figure 3 illustrates the recruitment process for the study. Potentially eligible individuals will be screened through either outpatient clinic or community settings. Research assistants will contact these individuals to provide a brief explanation of the study objectives and conduct a preliminary eligibility assessment. Those deemed potentially eligible will be invited to the nearest study site for confirmation of eligibility and a face-to-face baseline assessment.

All participants will be invited for follow-up assessments at 6 months and one year after completing the baseline assessments. Participants will receive travel allowance of 80–150 CNY (approximately 12–20 US dollars) for each visit, depending on the completion rate of assessments.

### Digital platform

Data in the DIMOSI are managed through the Zhiyoumian electronic data platform. Developed by the research team, this platform is equipped with capabilities for data collection, preliminary analyses, and management. All assessments in the study—including structured clinical interviews, validated questionnaires, cognitive tasks, sleep-activity diaries, anthropometric assessments, facial expression, and ecological momentary assessment (EMA)—for measuring mental disorders/symptoms, emotions, behaviors, cognitive function, sleep, activity, and other related parameters (Table 1) are integrated into this digital platform. Two apps, Zhiyoumian - Participant and Zhiyoumian - Researcher, were developed for both Android and IOS systems. The platform adopts end - to - end encryption technology to ensure data security.

**Table 1:** Assessments in each visit for the DIMOSI

Domain	Assessment approach	Measurement	Baseline visit	FU 1	FU 2
Mental disorders and symptoms	Clinical interview	Mental disorders and their Symptoms [29]	X	X	X
Sleep and Circadian Rhythm	Clinical interview	Diagnostic Interview for Sleep Patterns and Disorders [30, 31]	X	X	X
	Questionnaire	Insomnia Severity Index (ISI) [32, 33]	X	X	X
	Questionnaire	Morning and Evening Questionnaire 5 (MEQ-5) [34, 35]	X	X	X
	Questionnaire	Epworth Sleepiness Scale (ESS) [36, 37]	X	X	X
	Questionnaire	Ford Insomnia Response to Stress Test (FIRST) [38, 39]	X	X	X
	Questionnaire	Dysfunctional Beliefs and Attitudes about Sleep-16 (DBAS-16) [40, 41]	X	X	X
	Questionnaire	Multi-dimensional Fatigue Inventory (MFI) [42, 43]	X	X	X
	Questionnaire	Pre-sleep Arousal Scale (PSAS) [44]	X	X	X
	Questionnaire	Sleep Hygiene Practices Scale (SHPS) [45]	X	X	X
	Questionnaire	STOP-BANG questionnaire (SBQ) [46]	X		
	Questionnaire	Activity Diary (Sleep diary + Social Rhythm measure)	X X	X X	X X
	Monitoring devices	Wearable EEG	X X		X X
	Monitoring devices	Accelerometer	X X		
Emotion	Questionnaire	9-item Patient Health Questionnaire (PHQ-9) [47]	X	X	X
	Questionnaire	Generalized Anxiety Disorder Scale-7 (GAD-7) [48, 49]	X	X	X
	Questionnaire	Beck Scale for Suicide Ideation (BSI) [50, 51]	X	X	X
	Questionnaire	Positive and Negative Affect Schedule (PANAS) [52, 53]	X	X	X
	Questionnaire	Ruminative Responses Scale (RRS) [54, 55]	X		
Cognition	Questionnaire	Attention to Positive and Negative Information Scale (APNIS) [56, 57]	X	X	X
	Questionnaire	Barratt Impulsiveness Scale 11 (BIS-11) [58, 59]	X		
	Digital cognitive tasks	Psychomotor vigilance test (PVT)	X	X	X
	Digital cognitive tasks	Balloon Analogue Risk Task (BART)	X	X	X
	Digital cognitive tasks	Go/No-Go Task	X	X	X
	Digital cognitive tasks	N-Back task	X	X	X
physicality	Facial Recognition	Facial image	X	X	X
	Questionnaire	Short-form McGill Pain Questionnaire-2 (SF-MPQ-2) [60, 61]	X	X	X
	Questionnaire	Patient Health Questionnaire-15 (PHQ-15) [62, 63]	X	X	X
	Medical records	Height	X		
	Medical records	Weight	X		
Life events	Questionnaire	Quality of Life-BREF (QOL-BREF) [64]	X	X	X
	Questionnaire	Childhood Trauma Questionnaire (CTQ) [65, 66]	X		
	Questionnaire	Life Event Scale (LES) [67]	X	X	X
	Medical records	Medical history	X	X	X
	Medical records	Family history	X		
Life style	Questionnaire	Subjective daily pattern (activity, diet, and sleep)	X	X	X
Personality	Questionnaire	Big Five Inventory-2 (BFI-2) [68, 69]	X		
General Health	Ecological momentary assessment	EMA questionnaire, Supplementary Methods, Section 2	X X		
	Questionnaire	General information (Supplementary Methods, Section 1)	X	X	X

X delivered once; XX delivered every day for 7 days; XXX delivered every day according to the therapy

FU: Follow-up assessment

Both apps were evaluated using the System Usability Scale (SUS). The Cronbach’s α of Zhiyoumian–Participant app was 0.82 with a mean SUS score of 66.22 ± 13.41, from 610 recruited participants, while the Cronbach’s α of Zhiyoumian–Researcher app was 0.84 with a mean score of 72.31 ± 15.23, from 53 researchers of study sites, indicating acceptable usability for both applications.

Each participant will be asked to install Zhiyoumian - Participant, while all researchers will install Zhiyoumian - Researcher. Zhiyoumian - Researcher guides researchers through conducting structured clinical interviews based on the Diagnostic and Statistical Manual of Mental Disorders, Fifth Edition (DSM-5) [29] and the International Classification of Sleep Disorders, Third Edition (ICSD-3) [30] to determine the diagnoses of insomnia disorder, comorbidity, and other related information. Zhiyoumian – Participant integrates self-reported information, such as questionnaires and sleep-activity diaries, along with objective data from two wearable devices. Ecological momentary assessment(EMA), an intensive assessment method [70], will be administered four times daily over a consecutive seven-day period, assessing daily sleep, mood, physical activity, diet, and related parameters. Participants who responded to less than 30% of the prompts might be excluded from the EMA-specific analysis. It is designed to collect concise, real-time evaluations of key domains, including sleep, physical activity, emotion, and diet. The wearable EEG monitor (EEG Smart S1) and accelerometer (Axivity, AX3) will be provided to each participant to measure sleep parameters [71], physical activity [72], and other related physiological indicators [73, 74]. The data of the days when the Axivity is worn for less than 16 hours will be excluded. Participants will be included when meeting the criteria as wearing the device at least 16 hours in at least 3 days in the actigraphy analysis. Similarly, data from the wearable EEG monitor will be included only if at least 4 hours of scorable sleep data per night is available, and participants will be included in the analysis when meeting the criteria as wearing the device for at least 1 day. The digital platform benefits the real-time interaction between participants and researchers.

### Interviewer training and the management system

The DIMOSI fieldwork was conducted by a team consisting of sleep researchers, psychiatrists, research assistants, and nurses. A study manager based at Guangdong Mental Health Centre, Guangdong Provincial People’s Hospital (Guangdong Academy of Medical Sciences) oversaw the supervisors and their teams. Among the four experienced supervisors, two were PhD-level psychiatrists (with 17 and 13 years of clinical experience, respectively), one was a postdoctoral sleep researcher (with 5 years of clinical research experience), and one was an MA-level clinical mental health researcher (with 2 years of clinical research experience).

Each interviewer conducting clinical assessment for participants was required to hold at least a bachelor’s degree and 2 years of experiences in clinical research. Interviewers were also required to complete a 20-hour General Interviewer Training (GIT) course before working on any DIMOSI survey, which included text, video, and on-site observational learning. Potential interviewers were required to pass an interview test before being allowed to contact participants. They needed to successfully complete three independent interviews under the supervision of two professional DIMOSI interviewers and had to pass a stringent examination. Failure was defined as making 3 or more errors out of the 393 test items.

In the pilot phase, we invited two centres to participate in the platform testing, involving a total of 98 subjects throughout the research process. Based on the issues encountered during testing, we refined quality control details and optimized software functionality. To ensure the system meets the needs of both participants and researchers, ongoing monitoring of the platform’s acceptability and usability will be conducted, including monitoring the apps, assessment scheme, hardware measurement devices, e-CBTI platform, and web management platform. After the official start of the study, real-time monitoring of backend data was implemented and participants were contacted if data stream disruptions occurred from any device to determine whether issues were related to functionality or user experience. Researchers conduct weekly quality control assessments, and provide feedback to each centre.

**Fig. 4:**
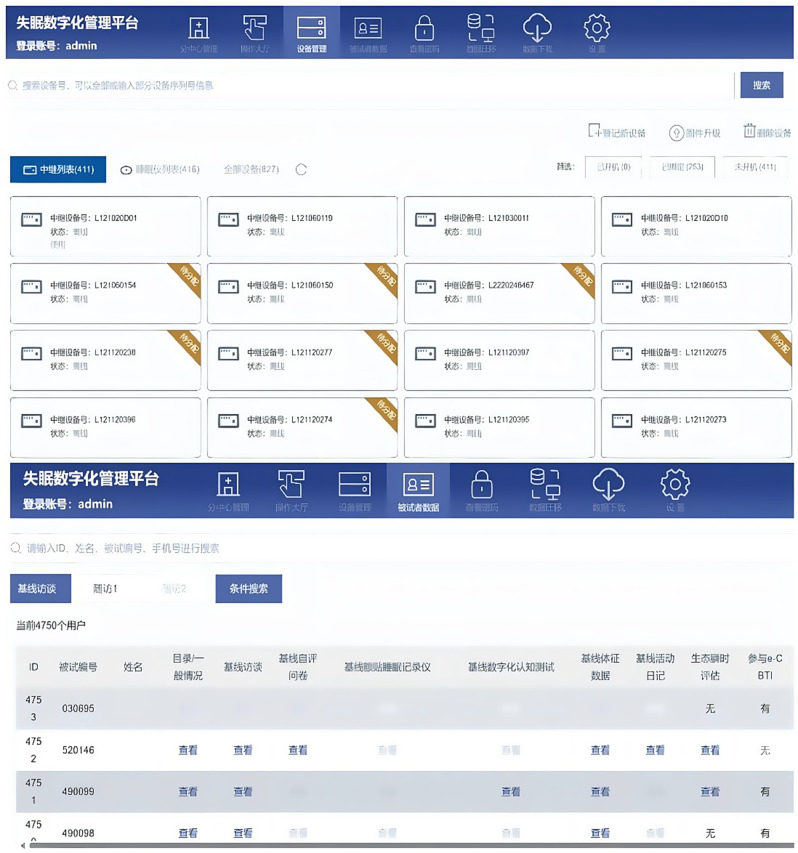
Administrative interface of the DIMOSI digital platform

Through monitoring of the collected data via the administrative interface, a data quality assessment is conducted using a data quality control checklist. The checklist covers aspects such as interview duration, interview logistics, physiological data quality, activity diary completion time, and the level of information completeness. Monitoring results are documented in a standardized data quality feedback form and provided as feedback to each centre. Figure 4 shows the administrative interface.

### Statistical analyses plan

#### Baseline descriptive analyses

The study will estimate descriptive statistics for demographic data, drop-out rates, and the number of participants using digital tools, ensuring no data loss or damage and maintaining data integrity throughout the research period. Multiple imputation will be applied to handle missing data if necessary.

#### Multimodal data integration

Questionnaire, EMA, wearable, and cognitive task data will be integrated using a unified preprocessing and clustering framework. Within each modality, features are selected to capture distinct aspects of insomnia-related functioning, such as mental disorders and symptoms, cognition, objective sleep parameters, and demographic characteristics. Variables with low variance or > 30% missingness will be excluded. To eliminate the influence of differing scales and units across features, continuous variables are standardized using Z-score normalization.

To address multicollinearity and redundant information, we construct a pairwise correlation matrix and visualized it with a heatmap to identify highly correlated feature pairs (|r| > 0.8). Two mainstream strategies will be considered: (1) Feature selection using Lasso regression with 10-fold cross-validation to retain the most informative variables while shrinking redundant coefficients to zero; and (2) Feature dimensionality reduction using principal component analysis (PCA), which transforms correlated variables into orthogonal principal components while preserving most of the data variance. After preliminary testing, PCA is selected as the final approach based on its superior performance in preserving global data structure and compatibility with K-means clustering’s independence assumptions. Components explaining > 85% of the cumulative variance will be retained for subsequent analyses.

#### Subtyping procedures

The standardized features from each modality are concatenated into a single feature matrix, and variance normalization is applied to balance the contribution of different modalities in subsequent analyses [75, 76]. PCA was further applied for dimensionality reduction, followed by K-means clustering to identify distinct insomnia subtypes.

The optimal number of clusters (k) was determined using the elbow, silhouette, and Gap statistics. To assess clustering robustness, bootstrap resampling (1,000 iterations) and consensus clustering will be performed [77]. Cluster characteristics will be summarized using standardized centroid differences and key feature importance, providing interpretable profiles of each subtype. Sensitivity analyses will be conducted to compare equal-weight versus data-driven weighting schemes to evaluate the robustness of the clustering results. The defining features of each cluster are examined to understand the underlying pathophysiological mechanisms and clinical presentations.

Aggregated features obtained from Zhiyoumian and wearable devices as well as their changes from baseline, will be incorporated into the statistical analyses. Machine learning techniques will be applied to build predictive models evaluating the stability and clinical predictive value of these subtypes.

#### Longitudinal modeling

We will use classification and regression methods to investigate whether baseline information can predict follow-up outcomes. Chi-square analysis, repeated measures analysis, and mixed-effects models will be employed to examine the relationships among the cross-sectional data and the changes over time across different visits. For binary outcomes (e.g., the occurrence of anxiety or depression), logistic regression models will be used, while linear regression models will assess continuous outcomes (e.g., changes in the Insomnia Severity Index (ISI) score). Mixed-effects models will analyze longitudinal changes while accounting for individual variability.

Statistical methods may be adjusted as needed based on the actual data, and we will adopt the most appropriate methods for the analyses. Any limitations in the analysis will be thoroughly addressed and elaborated upon in subsequent research publications.

## Results

In the current study, 127 researchers across study sites received consistent training in clinical interviews, the use of wearable devices, and other psychological assessments. We randomly selected 46 researchers to complete a reliability assessment for 8 clinical structural interviews. The Cochran’s Q values ranged from 29.2 to 43.0, with all p-values > 0.05, indicating a high consistency among interviewers.

From November 28, 2022, to June 30, 2025, approximately 5000 individuals with suspected insomnia disorder were screened. A total of 3134 participants were interviewed, of whom 2937 (93.7%) were recruited and 197 (6.3%) were excluded. Among those recruited, there are 1743 (59.3%) from outpatient clinics and 1194 (40.7%) from community settings. The most common reason for exclusion was psychotic symptoms resulting from other mental disorders, which led to the inability to complete the clinical interview. The recruitment procedure is shown in Fig. 3.

All participants met the ICSD-3 criteria for insomnia disorder, with a mean age of 37.3 ± 12.6 years and 1952 (66.5%) were females. At baseline, 2850 participants (97.1%) were suffering from current insomnia disorder as defined by the clinical interview based on ICSD-3. In terms of comorbidities, assessed by the structured clinical interviews administered via the Zhiyoumian-Researcher app, 505 participants (17.2%) had current depressive disorders, and 613 participants (20.9%) had current anxiety disorders. The mean score of the ISI was 15.5 ± 5.8, with a median (interquartile range) score of 16.0 (7.0). When comparing those from the community setting and clinic, we found that the participants from the clinic are significantly older than their counterparts. But there is no statistical difference in sex, comorbidities, and insomnia severity (Table 2).

**Table 2:** Demographics, comorbidities, and insomnia severity in participants from community and clinic settings（*N* = 2937）

	Community(*n* = 1194)	Clinic（*n* = 1743）	*p*
Age, mean±SD	35.9±12.3	38.2±12.3	＜0.001
Gender Female, n(%)	780 (65.3%)	1172 (67.2%)	0.283
Comorbidities, n(%)	364 (30.5%)	484 (27.8%)	0.115
Depressive Disorder	212(17.8%)	293(16.8%)	0.518
Generalized Anxiety Disorder	270(22.6%)	343(19.7%)	0.058
Bipolar Disorder	39(3.3%)	48(2.8%)	0.439
Panic Disorder	15(1.3%)	25(1.4%)	0.748
Post-Traumatic Stress Disorder	25(2.1%)	45(2.6%)	0.460
ISI, mean±SD	15.7±5.84	15.3±5.79	0.101
ISI ≥ 8, n(%)	1104(92.5%)	1602(92.9%)	0.625

Note: ISI, Insomnia Severity Index

In this EMA study, a total of 1231 participants (76.6%) provided eligible data for at least 50% of the time points, 1015 participants (63.2%) for at least 75%, and 746 participants (46.4%) for at least 90%. Furthermore, 2134 participants (72.7%) utilized accelerometers, while 2429 (82.7%) wore wearable EEG monitors for continuous assessments of their physical activity and sleep patterns. Of the 2850 participants with current insomnia, 2455 received e-CBTI treatment. Detailed data cleansing and analysis of the objective data will be reported elsewhere.

## Discussion

The DIMOSI study is a large-scale, national prospective cohort study on insomnia disorder in China. Several characteristics of the DIMOSI study, including its large sample size, prospective design, the integration of both subjective and objective assessments, and recruitment from both community and outpatient clinics across 33 centres in China, provide a unique opportunity to explore subtypes of insomnia disorder, as well as new prevention and intervention methods. All interviewers were well-trained and compliance with most assessments was satisfactory. Over 95% of participants had a current diagnosis of insomnia disorder, and nearly 20% had concurrent mood and/or anxiety disorders. The profile of the sample of the current study, for instance female dominance, is consistent with the previous epidemiological study [78, 79]. The majority of participants completed self-reported questionnaires, structured diagnostic interviews for mental and sleep disorders, cognitive function tasks, ecological momentary assessments, and continuous objective assessments of sleep and physical activity over a 7-day period.

This study is conducted on a digital multi-modal platform that integrates key research processes, including interviewer training and assessment, participant inclusion and exclusion criteria, ethical notifications, questionnaire assessments, cognitive task completion, and objective data collection. The platform is consistently used in both baseline and follow-up phases, ensuring a unified approach throughout the study. By utilizing this platform, the study maintains consistency in data collection across all stages, thereby enhancing both the quality and reliability of the research data. Additionally, all key aspects of data quality control are effectively managed within this digital platform. The application of the digital platform not only significantly improves research efficiency but also ensures consistency in operational processes at every stage, minimizing manual errors to the greatest extent possible. The use of the digital platform facilitates the accurate collection of multi-modal data from the real world and enables the integration and analysis of large datasets using advanced algorithms.

Research has found that insomnia disorder is a 24-hour condition [80], encompassing not only nighttime sleep disturbances but also daytime cognitive decline and alterations in circadian and activity patterns [81–83]. In our study, we used accelerometers and wearable EEG devices as objective assessment tools to measure sleep quality and rest-activity patterns. We did not use PSG because it is time-consuming and labor-intensive, and one single-night PSG assessment may not adequately capture the day-to-day variations in sleep conditions. We did not include biological sample (e.g., cortisol and melatonin) tests because of lacking reliable biological markers for insomnia disorders currently. Excluding biological samples does not affect our ability to test the research hypotheses.

This promising insomnia cohort, with a target sample size of 4,000, aims to provide sufficient statistical power for subtyping insomnia disorder. Additionally, we collect a series of objective multimodal data. Compared to previous studies using accelerometers to assess the sleep phenotype [84, 85], the current study offers insights into clinical and subthreshold symptoms, as well as cognitive functions [86, 87], which have potential clinical implications. In addition, the current cohort has the potential to clarify the associations of insomnia with other medical conditions and their underlying shared mechanism, enhancing the identification of at-risk individuals and prevention development. For example, given the established bidirectional relationship between insomnia and suicidality, the identification of distinct insomnia subtypes holds significant broader implications [88, 89]. Therefore, this study is likely to fill the knowledge gap in the understanding of insomnia disorder, particularly in the area of clinical subtyping.

## Strengths and limitations

The DIMOSI study has several notable strengths. First, the current study is a prospective cohort with a nationally representative large-scale sample recruited from both the community and clinical settings. All participants met diagnostic criteria through face-to-face clinical interviews based on ICSD-3 standards. This provides a sufficient number of representative participants, enabling the exploration of systematic subtypes and the assessment of their stability and predictive value. Second, the study employs comprehensive assessments, including traditional subjective measures, cognitive function evaluations, and objective measures of sleep and physical activity. Third, the integrated digital platform broadens the application scenarios in terms of time and location, making assessments of insomnia disorder more convenient and improving participant compliance.

However, several limitations of this study should be acknowledged. First, some participants were using sleep medications at baseline, which may have resulted in lower ISI scores at enrollment. We recorded these conditions and used statistical techniques to control for these factors. Second, considering that the main aim of this study is to analyze the subtype analysis of insomnia disorders rather than biological markers, biological sample testing was not included [90]. Third, while the comprehensive application of digital technology has conferred efficiency and high data quality, it may exclude some patients who are unable to use electronic devices. Finally, the current study uses convenience sampling, which may have introduced selection bias and limited generalizability, but we mitigated this via screening logs and community-clinic sensitivity analyses.

## Conclusions

The DIMOSI study is a national multicentre prospective cohort study utilizing an integrated digital assessment platform, conducted among adult patients with insomnia disorder. This study provides a unique opportunity to explore the subtypes of insomnia disorder, which hold predictive value for the clinical trajectories and treatment responses.

## Electronic supplementary material

Below is the link to the electronic supplementary material.

Supplementary Material 1

## Data Availability

The datasets generated and analysed during the current study are not publicly available due to participant privacy and institutional regulations, but are available from the corresponding author on reasonable request and with appropriate institutional approval.

## Abbreviations

DIMOSI: The Digital Multi-modal Approaches to Subtyping Insomnia Disorder
DIS: Difficulty initiating sleep
DMS: Difficulty maintaining sleep
EPV: Estimated events per variable
e-CBTI: Electronic Cognitive Behavioral Therapy for Insomnia
EMA: Ecological momentary assessment
ICSD-3: The International Classification of Sleep Disorders, Third Edition
DSM-5: The Diagnostic and Statistical Manual of Mental Disorders, Fifth Edition
