# Supplementary material for: Digital multi-modal approaches to subtyping insomnia disorder (DIMOSI): study design, rationale, digital platform, and preliminary baseline characteristics of a national prospective cohort study
Source: BMC Psychiatry. 2025 Dec 10;26:74. doi: 10.1186/s12888-025-07648-9 (PMC12849073; doi:10.1186/s12888-025-07648-9)
Supplement: Supplementary file 1 — Supplementary Material 1 [file 12888_2025_7648_MOESM1_ESM.docx]

**Supplementary material**

**Title: Digital multi-modal approaches to subtyping insomnia disorder (DIMOSI): study design, rationale, digital platform, and preliminary baseline characteristics of a national prospective cohort study**

| Supplementary Methods, Section 1 | Detailed information on general information | 2 |
| --- | --- | --- |
| Supplementary Methods, Section 2 | Detailed information on EMA self-assessment module | 8 |

**Supplementary Methods, Section 1. Detailed information on general information**

**1. Basic Information**

1.1 (Reserved for additional items if needed)

1.1 Full Name: ________________________

1.2 Date of Birth: ____ Year ____ Month ____ Day

1.3Ethnicity: ________________________

1.4 Sex: (1□ Male 2□ Female)

1.5 Permanent Residence: ____ Province ____ City

1.6 Residence Type: (1□ Urban 2□ Rural)

1.7 Educational Level: 1□ Primary School or Below 2□ Junior High School, Senior High School, or Technical Secondary School 3□ Junior College, Bachelor's Degree, or Above

1.8 Marital Status: 1□ Unmarried 2□ Married 3□ Divorced 4□ Widowed 5□ Other ______

1.9 Living Alone: 0□ No 1□ Yes

1.10 Occupation

1.10.1 Occupation Type: 1□ Paid Employment (Skip to 10.2) 2□ Full-time Homemaker 3□ Student 4□ Retired

1.10.2 Work Schedule: ____ Years of Work Experience; Daily Work Start Time: ______; Daily Work End Time: ______

1.10.3 Shift Work Requirement: 0□ No 1□ Yes; Shift Nature: ______ ① Overnight Shift (8:00 PM – 8:00 AM) ② Midnight Shift (12:00 AM – 8:00 AM) ③ Flexible Shifts

1.11 Personal Monthly Income: 1□ ≤ 5,000 CNY 2□ 5,001 - 10,000 CNY 3□ 10,001 - 50,000 CNY 4□ ＞ 50,000 CNY (Unemployed individuals or full-time homemakers should fill in the average monthly income per family member)

**2. Lifestyle History (Please answer based on your situation in the past 3 months)**

| Behavior | Never | Rarely (＜1 time/week) | Moderately (1-2 times/week) | Frequently (≥3 times/week) |
| --- | --- | --- | --- | --- |
| a) Drinking Tea | 0 □ | 1 □ | 2 □ | 3 □ |
| b) Drinking Coffee | 0 □ | 1 □ | 2 □ | 3 □ |
| c) Drinking Energy Drinks (e.g., Red Bull) | 0 □ | 1 □ | 2 □ | 3 □ |
| d) Drinking Alcohol | 0 □ | 1 □ | 2 □ | 3 □ |
| e) Smoking | 0 □ | 1 □ | 2 □ | 3 □ |
| f) Substance use (e.g., Cocaine) | 0 □ | 1 □ | 2 □ | 3 □ |

**3. Sleep Status (Please answer based on your situation in the past 3 months; "usually" refers to occurring 3 times or more per week)**

3.1 Do you usually sleep in a separate room by yourself? (0□ No 1□ Yes)

3.2 Do you usually sleep in a separate bed by yourself? (0□ No 1□ Yes)

3.3 What time do you usually go to bed at night? (Work/School Days: ____ Hour ____ Minute; Rest Days: ____ Hour ____ Minute)

3.4 What time do you usually wake up every day? (Work/School Days: ____ Hour ____ Minute; Rest Days: ____ Hour ____ Minute)

3.5 Do you usually take a nap? (0□ No 1□ Yes ____ ① Lie in bed without sleeping ② 0-1 Hour ③ More than 1 Hour)

3.6 Do you easily wake up after falling asleep at night? (0□ No 1□ Yes; Usually ____ Times/Night)

3.7 Do you have the habit of getting up at night (to use the toilet, etc.)? (0□ No 1□ Yes; Usually ____ Times/Night)

3.8 How many hours of sleep do you think you need to feel well-rested? ______ Hours

3.9 Do you think you get enough sleep? (0□ No 1□ Yes)

3.10 Are you currently experiencing insomnia? (0□ No 1□ Yes; Duration: ____ Months; Frequency: ____ Times/Week)

3.11 What do you usually do 1 hour before going to bed at night? (Multiple selections allowed)

| 1□ Studying/Working | 2□ Watching TV Programs | 3□ Exercising | 4□ Making Phone Calls | 5□ Eating |
| --- | --- | --- | --- | --- |
| 6□ Using Electronic Devices | 7□ Reading Paper Books/Newspapers | 8□ Resting | 9□ Other: _________ | 9□ Other: _________ |

3.12 Do you have the habit of listening to audio broadcasts to help you fall asleep? (0□ No, skip directly to 3.15 1□ Yes)

3.12.1 How often do you usually listen to audio broadcasts before bed? 1□ ＜1 time/month; 2□ 1-3 times/month; 3□ 1-2 times/week; 4□ ≥3 times/week

3.12.2 How long do you usually listen to audio broadcasts each time before bed? ______ Hours

3.12.3 What type of audio broadcasts do you usually choose before bed? (Multiple selections allowed)

| 1□ Music | 2□ Entertainment (Talk Shows, Cross-talk, etc.) | 3□ News | 4□ Foreign Language Content | 5□ Other |
| --- | --- | --- | --- | --- |

3.12.4 Do you think listening to audio broadcasts before bed helps with your sleep? (0□ No 1□ Yes)

3.15 Do you have the habit of using fragrance to help you fall asleep? (0□ No, skip directly to Section 4 1□ Yes)

3.15.1 Do you think fragrance helps with your sleep? (0□ No 1□ Yes)

**4. Activity Status**

4.1 On average, how much time do you spend on the following activities per day? (Please answer based on your situation in the past 3 months)

| a. Studying/Working: ________ Hours | b. Watching TV Programs: ________ Hours | c. Physical Activities: ________ Hours |
| --- | --- | --- |
| d. Commuting/Transportation: ________ Hours | e. Reading Paper Books/Newspapers: ________ Hours | f. Making Phone Calls: ________ Hours |
| g. Hobbies (Excluding Physical Activities and Reading, e.g., Playing the Piano, Painting, Dancing, etc.): ________ Hours | g. Hobbies (Excluding Physical Activities and Reading, e.g., Playing the Piano, Painting, Dancing, etc.): ________ Hours | g. Hobbies (Excluding Physical Activities and Reading, e.g., Playing the Piano, Painting, Dancing, etc.): ________ Hours |
| h. Total Time Spent on Phone/Tablet/Computer: ________ Hours | h. Total Time Spent on Phone/Tablet/Computer: ________ Hours | h. Total Time Spent on Phone/Tablet/Computer: ________ Hours |

**5. Medical History (Unless specified otherwise, answer the following questions based on your situation in the past year; multiple selections allowed)**

**5.1 Medical Conditions**

a. Sleep Disorders: 0□ None 1□ Sleep Disordered Breathing 2□ Restless Legs Syndrome 3□ Rapid Eye Movement Sleep Behavior Disorder 4□ Other ______

b. Cardiovascular Diseases: 0□ None 1□ Hypertension 2□ Coronary Heart Disease 3□ Atrial Fibrillation 4□ Other ______________________________

c. Endocrine Diseases: 0□ None 1□ Diabetes Mellitus 2□ Hyperthyroidism 3□ Hypothyroidism 4□ Other ____________

d. Digestive System Diseases: 0□ None 1□ Gastroesophageal Reflux Disease 2□ Gastric/Duodenal Ulcer 3□ Irritable Bowel Syndrome 4□ Other ______

e. Nervous System Diseases: 0□ None 1□ Stroke 2□ Parkinson's Disease 3□ Alzheimer's Disease 4□ Encephalitis 5□ Other ___________

f. Autoimmune Diseases: 0□ None 1□ Hashimoto's Thyroiditis 2□ Systemic Lupus Erythematosus 3□ Rheumatoid Arthritis 4□ Other _____

g. Infectious Diseases: 0□ None 1□ Hepatitis B 2□ Hepatitis C 3□ Tuberculosis 4□ Other ___________________________________

h. Skin Diseases: 0□ None 1□ Herpes Zoster 2□ Eczema 3□ Urticaria 4□ Severe Acne 5□ Psoriasis 6□ Other _________

i. Malignant Tumors: 0□ None 1□ Yes ______________________________________________________________

j. Chronic Pain: 0□ None 1□ Headache 2□ Trigeminal Neuralgia 3□ Fibromyalgia 4□ Postherpetic Neuralgia 5□ Cancer-Related Pain 6□ Postoperative or Post-Traumatic Pain 7□ Other _______________________________________

k. Mental Disorders (Select if you have ever been diagnosed with the following diseases, not limited to the past year): 0□ None 1□ Anxiety Disorder 2□ Depression 3□ Bipolar Disorder 4□ Panic Disorder 5□ Schizophrenia 6□ Post-Traumatic Stress Disorder 7□ Other _____________

l. Other Diseases: 0□ None 1□ Yes □ ____________________________________________________________

5.2 Medications You Are Currently Taking: 0□ None □ 1□ Yes, please specify the names of the medications: ____________________________________

5.3 Have you had any surgical history in the past 3 months (excluding superficial subcutaneous surgeries)? 0□ No 1□ Yes ___________________________

**6. Family History (If you are unsure about the following situations, select "No")**

6.1 Do either of your parents have a history of chronic insomnia (characterized by difficulty falling asleep, difficulty maintaining sleep, or early awakening, occurring more than 3 days per week for 3 months or longer)? (0□ No/Unsure 1□ Yes __ ① Father ② Mother ③ Both Parents)

6.2 Do your parents consistently go to bed much later than desired (e.g., after 1:00 AM or more than 2 hours later than the desired bedtime) and have obvious "night owl" traits (e.g., more alert in the evening than in the morning)? (0□ No/Unsure 1□ Yes __ ① Father ② Mother ③ Both Parents)

**7. Menstrual History (For Females Only)**

7.1 Menstrual Status:

| 1□ Premenopausal |
| --- |
| 2□ Perimenopausal (Around menopause; irregular cycles but at least one period in the past 12 months) |
| 3□ Postmenopausal; Age at Last Menstruation: _______ Years Old |
| 4□ Never Had Menstruation |

7.2 Age at Menarche (First Menstruation): _________ Years Old

7.4 Are your menstrual cycles regular? (If menstruation has stopped, were your cycles regular before cessation?) 0□ No 1□ Yes

7.5 Length of Menstrual Cycle: _________ Days

7.6 Do you have dysmenorrhea? 0□ No 1□ Yes

7.7 Have you given birth to a child (including induced abortion and stillbirth)? 0□ No (Skip directly to the ISI Scale Section) 1□ Yes (Number of Children: ______ )

7.7.1 Are you currently troubled by postpartum sequelae? (Multiple selections allowed) 0□ None 1□ Pelvic Organ Prolapse (Including Uterine Prolapse, Anterior or Posterior Vaginal Wall Prolapse) 2□ Postpartum Urinary Incontinence 3□ Diastasis Recti 4□ Symphysis Pubis Pain 5□ Other ______

**Supplementary Methods, Section 2. Detailed information on EMA self-assessment module**

The EMA self-assessment is conducted **4 times a day** at 8:00, 13:00, 18:00, and 23:00 (adjustments can be made based on the participant’s daily schedule) for **7 consecutive days**. Some questions only need to be completed at 8:00 or 23:00.

**1. Morning and Sleep Behavior (to be completed only at 8:00)**

How well-rested did you feel upon waking up?

| 1 Not at all rested | 2 | 3 | 4 | 5 | 6 | 7 Extremely well-rested |
| --- | --- | --- | --- | --- | --- | --- |

Which of the following sleep problems, if any, did you experience last night? (Select all that apply)

| Difficulty falling asleep | Nighttime awakenings | Waking up too early | Nightmares | Other sleep problems | None |
| --- | --- | --- | --- | --- | --- |

Did you take any sleep medication last night?

| No | Yes |
| --- | --- |

**2. Context of Assessment (Environment, Social Contact)**

What are you doing right now?

| Doing nothing / Waiting for something |
| --- |
| Napping / Resting |
| Eating |
| Doing housework |
| Working (paid or unpaid) |
| Shopping |
| Exercising |
| Walking the dog |
| Traveling or on a business trip |
| Watching TV |
| Listening to music |
| Making a phone call |
| Having a face-to-face conversation |
| Leisure activities (non-exercise) |
| Others |

**3. Emotional and Physical States (Mood Circumplex)**

How happy (or sad) do you feel right now?

| 1 Extremely happy | 2 | 3 | 4 | 5 | 6 | 7 Extremely sad |
| --- | --- | --- | --- | --- | --- | --- |

How relaxed (or nervous) do you feel right now?

| 1 Extremely relaxed | 2 | 3 | 4 | 5 | 6 | 7 Extremely nervous |
| --- | --- | --- | --- | --- | --- | --- |

How energetic do you feel right now?

| 1 Extremely inactive | 2 | 3 | 4 | 5 | 6 | 7 Extremely active |
| --- | --- | --- | --- | --- | --- | --- |

How energetic (or tired) do you feel right now?

| 1 Extremely tired | 2 | 3 | 4 | 5 | 6 | 7 Extremely energetic |
| --- | --- | --- | --- | --- | --- | --- |

How focused do you feel right now?

| 1 Extremely focused | 2 | 3 | 4 | 5 | 6 | 7 Extremely unfocused |
| --- | --- | --- | --- | --- | --- | --- |

How irritable (or angry) do you feel right now?

| 1 Not at all irritable / angry | 2 | 3 | 4 | 5 | 6 | 7 Extremely irritable / angry |
| --- | --- | --- | --- | --- | --- | --- |

How fast are your thoughts right now?

| 1 Extremely fast, many thoughts | 2 | 3 | 4 | 5 | 6 | 7 Extremely slow, mind is blank |
| --- | --- | --- | --- | --- | --- | --- |

How much enjoyment do you feel from things right now?

| 1 Feel extremely enjoyable | 2 | 3 | 4 | 5 | 6 | 7 Feel no enjoyment at all |
| --- | --- | --- | --- | --- | --- | --- |

Compared to your usual self, how restless do you feel right now?

| 1 No restlessness at all | 2 | 3 | 4 | 5 | 6 | 7 Extremely restless, unable to sit still |
| --- | --- | --- | --- | --- | --- | --- |

How hungry do you feel right now?

| 1 Not hungry at all | 2 | 3 | 4 | 5 | 6 | 7 Extremely hungry |
| --- | --- | --- | --- | --- | --- | --- |

**4. Activities**

Since the last assessment (or since waking up), have you napped or rested?

| No | Yes |
| --- | --- |

How long did your nap / rest last?

| Less than 30 minutes | 30-60 minutes | 1-2 hours | More than 2 hours |
| --- | --- | --- | --- |

Please select the intensity level of your activities since completing the last questionnaire (or since waking up this morning).

| Strenuous: e.g., running, cycling (high speed), lifting heavy objects, shoveling, etc. |
| --- |
| Moderate: e.g., playing tennis, cycling at a moderate pace, carrying groceries, etc. |
| Light: e.g., walking, climbing stairs, daily household chores, etc. |

Since the last assessment (or since waking up this morning), how many minutes did you spend on strenuous exercise or heavy physical activity?

| Approx. 5 minutes or less | Approx. 10 minutes | Approx. 20 minutes | Approx. 30 minutes | Approx. 40 minutes | Approx. 50 minutes | 1 hour or more |
| --- | --- | --- | --- | --- | --- | --- |

Since the last assessment (or since waking up this morning), how many minutes did you spend on moderate activities (making you breathe slightly heavier than usual)?

| Approx. 5 minutes or less | Approx. 10 minutes | Approx. 20 minutes | Approx. 30 minutes | Approx. 40 minutes | Approx. 50 minutes | 1 hour or more |
| --- | --- | --- | --- | --- | --- | --- |

Since the last assessment (or since waking up this morning), how many minutes did you spend on light activities (breathing about the same as usual)?

| Approx. 5 minutes or less | Approx. 10 minutes | Approx. 20 minutes | Approx. 30 minutes | Approx. 40 minutes | Approx. 50 minutes | 1 hour or more |
| --- | --- | --- | --- | --- | --- | --- |

**5. Food, Drink, Substances**

Since the last assessment (or since waking up this morning), have you consumed any of the following?

| Caffeinated drinks (e.g., coffee, tea, cola, etc.) | Alcoholic drinks (e.g., red wine, beer, spirits, etc.) | None |
| --- | --- | --- |

How many caffeinated drinks did you consume? (If applicable)

| 0 | 1 cup | 2 cups | 3 cups | 4 cups | 5 cups or more |
| --- | --- | --- | --- | --- | --- |

How many alcoholic drinks did you consume? (If applicable)

| 0 | 1 glass/cup | 2 glasses/cups | 3 glasses/cups | 4 glasses/cups | 5 glasses/cups or more |
| --- | --- | --- | --- | --- | --- |

Since the last assessment (or since waking up this morning), how many times have you eaten?

| 0 | 1 time | 2 times | 3 times | 4 times | 5 times or more |
| --- | --- | --- | --- | --- | --- |

How much did you eat?

| Nothing | Only snacks | A light meal | A regular meal | A very large meal |
| --- | --- | --- | --- | --- |

How many cigarettes have you smoked?

| 0 | 1 | 2 | 3 | 4 | 5 | 6-10 | 11-20 | More than 20 |
| --- | --- | --- | --- | --- | --- | --- | --- | --- |

**6. Daily Events and Overall Health (to be completed only at 23:00)**

Please recall your experience over the entire day (not since completing the last questionnaire) to answer the following questions.

How would you rate your physical health today?

| 1 Extremely poor | 2 | 3 | 4 | 5 | 6 | 7 Extremely good |
| --- | --- | --- | --- | --- | --- | --- |

Did you experience any of the following problems today? (Check all that apply)

| Allergies |
| --- |
| Asthma or difficulty breathing |
| Digestive, intestinal, or stomach problems |
| Muscle/joint pain |
| Rapid or pounding heartbeat |
| Headache or migraine |
| Dizziness, feeling lightheaded |
| Head hit or injury |

Did you feel a lack of energy for most of the day, as if you were carrying a heavy load, or as if your hands and feet felt heavy?

| No | Yes |
| --- | --- |

**7. Pain and Headache**

Are you experiencing any pain right now?

| 1 No pain at all | 2 | 3 | 4 | 5 | 6 | 7 Severe pain |
| --- | --- | --- | --- | --- | --- | --- |

**8. Menstruation and Saliva Sampling Task (Optional Section) (to be completed only at 23:00)**

Are you currently menstruating ?

| No | Yes |
| --- | --- |
